# Impact of a Nonfatal Dengue Episode on Disability-Adjusted Life Years: A Systematic Analysis

**DOI:** 10.4269/ajtmh.18-0309

**Published:** 2018-10-01

**Authors:** Wu Zeng, Yara A. Halasa-Rappel, Laure Durand, Laurent Coudeville, Donald S. Shepard

**Affiliations:** 1 Heller School for Social Policy and Management, Brandeis University, Waltham, Massachusetts;; 2 Sanofi Pasteur, Lyon, France

## Abstract

As dengue causes about 4,000 symptomatic nonfatal episodes for every dengue death globally, quantitative disability assessments are critical to assess the burden of dengue and the cost-effectiveness of dengue control interventions. This systematic analysis of disability or quality of life lost from a symptomatic nonfatal dengue episode combined a systematic literature review, statistical modeling, and probabilistic sensitivity analyses. We conceptualized a dengue episode as having two phases: acute and persistent symptoms. Our estimates for the acute phase, consisting of onset and recovery periods and defined as the first 20 days (0.054 year), were based on literature review. We searched PubMed, POPLINE, EconLit, Google Scholar, scientific conferences, and other sources, for “dengue” plus “quality of life” or related terms. From 4,322 initial entries, six met our criteria (original studies with empirical data). The median disability-adjusted life year (DALY) burden for the acute phase was 0.011 (95% certainty interval [CI]: 0.006–0.015) for ambulatory episodes, 0.015 (CI: 0.010–0.020) for hospitalized episodes, and 0.012 (CI: 0.006–0.019) overall. Using literature reviews about persistent dengue, we estimated that 34% of episodes experienced persistent symptoms with a median duration of symptoms of 0.087 (CI: 0.040–0.359) year, which resulted in median DALYs of 0.019 (CI: 0.008–0.082). Thus, the overall median DALY burden was 0.031 (CI: 0.017–0.092) for ambulatory episodes, 0.035 (CI: 0.024–0.096) for hospitalized episodes, and 0.032 (CI: 0.018–0.093) overall. Our dengue-specific burden of a dengue episode was 2.1 times the 2013 Global Burden of Disease estimate. These literature-based estimates provide an empirical summary for policy and cost-effectiveness analyses.

## INTRODUCTION

Dengue is one of the most common mosquito-transmitted diseases, placing about four billion people globally at risk^1^ and causing substantial impairment of population health globally. Between 1990 and 2013, an estimated average of 9,221 dengue deaths occurred annually, with 58.4 million apparent dengue cases in 2013. The combination of premature deaths due to dengue and acute dengue episodes was responsible for 1.14 million disability-adjusted life years in 2013.^2^

An accurate estimate of the disease burden of dengue relies on accurate epidemiological data on incidence, prevalence, mortality rate, and accurate estimates of quality-adjusted life years (QALYs)^3^ or disability-adjusted life years (DALYs) per episode in different settings for different severities of dengue cases. Both QALYs and DALYs combine morbidity and mortality, the duration of illness, and quality of life (QoL) or disability for the illnesses. A QALY constitutes 1 year of good health gained whereas a DALY represents 1 year of good health lost.^4^ Subsequently, the QALY has become the principal measure of the health improvements resulting from an intervention, and the DALY has become the main measure of the burden of a disease or risk factor.

Key elements of the burden of dengue studies and the cost-effectiveness of interventions against dengue are estimates of the level of disability during each day affected by dengue and the duration of a dengue episode. These estimates of the impact of dengue on QoL, which can be quantified by QALYs or DALYs, can be used to measure the gain from vaccination and other interventions. To our knowledge, no consensus or systematic review exists concerning the impact of a dengue episode on QALYs gained or DALYs lost.

The best known approximation is part of the 2013 Global Burden of Diseases (GBD) studies.^2^ Global Burden of Diseases found that each dengue episode created an average 0.0097 DALYs; most (89.9%) of this burden was attributed to persistent symptoms. However, as the GBD required a standardized approach for 301 conditions, it assessed the disability during the acute phase only for a generic infectious disease, not specifically for dengue. However, symptomatic dengue cases differ from many acute infectious diseases in several ways. First, in addition to fever and malaise, dengue, sometimes called “bone break fever,” may cause severe pain,^5^ which significantly lowers patients’ QoL. Second, dengue may persist for months with chronic symptoms, such as fatigue or depression.^6,7^ Third, adults in endemic areas are aware that a dengue case can become life threatening within a few hours if it causes plasma leakage, adding fear to the physical distress.

A recent review has assessed the prevalence of persistent or chronic dengue but not its disease burden.^8^ This study aims to fill the gap by conducting a systematic review of QALYs or DALYs affected by an acute dengue episode and converting the prevalence of persistent dengue into its disease burden. Thus, this study estimates the disease burden per nonfatal dengue episode, which can provide necessary information for economic evaluation of dengue vaccinations, based on a systematic review of disability weights, QoL, and duration of dengue illness.

## METHODS

### Search strategy for acute episode.

To identify articles for review, we searched for “dengue” combined with “quality of life” or a similar term. These terms were disability, QALY, DALY, or foreign equivalents.

We searched five major bibliographic databases on public health and economics from 1980 through 2017: 1) Cochrane Database of Systematic Reviews, 2) EMBASE Global Health Library-Regional databases; 3) PubMed/MEDLINE databases; 4) databases for economics literature, including RePEc, EconPapers, and EconLit; and 5) abstracts from two annual conferences focusing on outcome evaluations and tropical medicine—the American Society of Tropical Medicine and Hygiene and the International Society for Pharmacoeconomics and Outcomes Research. Because of the absence of a comprehensive database for conferences and the expectation that useful data from older abstracts would have been published, we limited the conference search to 2016 and 2017. We also conducted a search for grey literature through the POPLINE database. To include more grey literature for review, we also searched through Google Scholar using the same key words.

### Inclusion and exclusion criteria.

All the search records were first uploaded in Endnote X8 (Clarivate Analytics, Philadelphia, PA) and screened by Wu Zeng. The non-duplicate records were first reviewed through titles and abstracts to assess their relevance and eligibility by satisfying all three criteria: 1) the study title and abstract or executive summary was available in English, French, Portuguese, or Spanish; 2) the full study was published or released from 1980 through 2017, or the conference abstract was published in 2016 or 2017; and 3) the study reported empirical findings about the disability or QoL associated with symptomatic dengue infections. Studies lacking original numerical assessment of the degree of disability or merely using previously generated estimates were excluded.

### Acceptability assessment.

We assessed the acceptability of each article based on the appropriateness of its methods and likely validity of the data (Supplemental Appendix Table 1). The assessment examined the sampling and representativeness of the target population, the proportion of the original sample with valid data for analysis, appropriateness of tools and procedures used for measuring DALYs and QALYs, appropriateness of analysis and interpretation of results and their implications, and generalizability of results.

### Data extraction from the literature.

We created an Excel template to record study characteristics, sample demographics, quality attributes, and outcome measures. The study characteristics recorded the country in which the data were collected and type of patient recruitment (e.g., type of health-care institution). The sample demographics confirmed dengue patients’ characteristics, including age, sample size, and setting (i.e., hospitalized versus ambulatory cases). Outcome measures included QoL or disability, duration of illness, the day when the worst QoL occurred, and measurement tools used to assess QoL or disability.

### Overall framework of calculating disability burden (DB).

**Figure 1.**
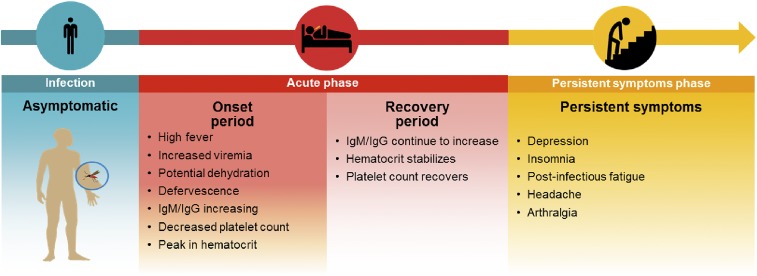
Course of a dengue infection showing the acute and persistent symptoms phases. Symptoms and quality of life generally vary over a dengue episode. IgM/IgG = immunoglobin M/immunoglobin G.

Using the conceptual framework of cost-effectiveness or cost-utility analysis,^3^ where the QoL associated with perfect health and death are, respectively, one and zero, the DB in DALYs for a nonfatal dengue episode is the sum of the QoL for each day during the dengue episode. This is represented mathematically by Eq. (1):

$$\text{DB}=\int_{0}^{T}\left[1-q\left(t\right)\right]\text{dt},$$ (1)

where *q*(*t*) is QoL at time *t* and *T* is the maximum duration of symptoms. Both *t* and *T* are expressed as fractions of a year. To apply Eq. (1), we must examine the evolution of QoL over time, denoted by the mathematical function *q*(*t*). After a dengue infection, a symptomatic dengue episode is composed of two phases: the acute phase that affects all patients and a persistent symptoms phase that affects a fraction of patients developing this disease (see Figure 1). The acute phase is further subdivided into onset and recovery periods. We present below successively the assessment performed for each of these phases and periods.

### Disability-adjusted life years during the acute phase.

**Figure 2.**
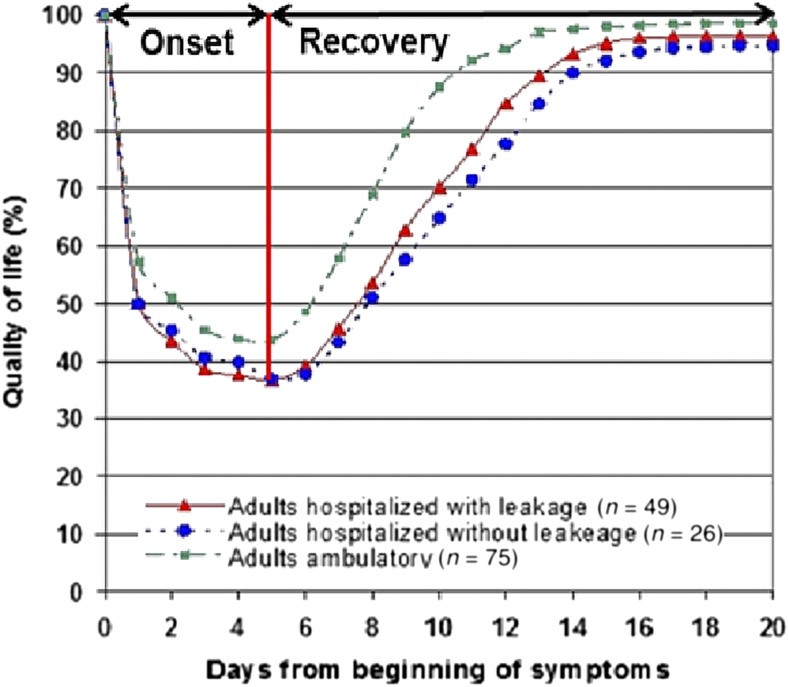
Quality of life for ambulatory and hospitalized dengue cases during the acute phase. This diagram, adapted from Lum et al.^9^, shows the 20-day (0.055 year) acute phase of a dengue episode, consisting of the onset (5 days or 0.014 year) and recovery (15 days or 0.041 year) periods. This figure appears in color at www.ajtmh.org.

The most thorough empirical data on the DB of an acute dengue episode come from Lum et al.,^9^ who reported the QoL of symptomatic dengue by day from days 1 through 20 from the onset of illness (see Figure 2). In their study, patients experienced a drastic decline in QoL during the onset period. Testing alternative power functions, we found that their evolution of QoL was best represented by a power function of order five. At the end of this recovery period, a fraction of patients continued to experience symptoms. The evolution of QoL during the recovery period was more gradual and was best represented by two functions. The QoL improved following an S-shape function until it stabilized. We termed the time from the end of the acute phase until the QoL stabilized the initial recovery subperiod and the subsequent stable period through day 20 the remaining recovery subperiod. The patient’s QoL during the remaining recovery subperiod was best represented by a constant function.

In Eq. (2) we use A to represent the entire acute phase of the dengue episode. Based on Lum et al.,^9^ we consider the entire acute phase to be 20 days. Equation (2) sums the burden across the onset and recovery periods over the entire acute phase (20 days), denoted by DB_A_. DB_A_ includes the entire acute phase (the onset period, the initial recovery period, and the remaining recovery period).

$${\text{DB}}_{\text{A}}=\int_{0}^{t_{\text{O}}}\left[\left[1-q\left(t_{\text{O}}\right)\right]\left[1-{\left(\frac{t_{\text{O}}-t}{t_{\text{O}}}\right)}^{5}\right]\right]\text{dt}+\int_{t_{\text{O}}}^{t_{\text{O}}+t_{\text{R}}}\left[1-q\left(t_{\text{O}}\right)-\frac{q\left(t_{\text{O}}+t_{\text{R}}\right)-q\left(t_{\text{O}}\right)}{t_{\text{R}}}\left(t-t_{\text{O}}\right)\right]\text{dt}+\int_{t_{\text{O}}+t_{\text{R}}}^{t_{\text{A}}}\left[1-q\left(t_{\text{O}}+t_{\text{R}}\right)\right]\text{dt},$$ (2)

Here $t_{\text{O}},t_{\text{R}},t_{\text{A}},q\left(t_{\text{O}}\right),q\left(t_{\text{O}}+t_{\text{R}}\right)$ correspond to the duration of the onset (O) period, duration of the recovery (R) period, duration of the entire acute (A) episode, the QoL at *t*_O_ (i.e., the lowest QoL during the dengue episode), and at *t*_O_ + *t*_R_ (i.e., the QoL at the end of the recovery period), respectively. The duration variables *t*_O_, *t*_R_, and *t*_A_ are expressed as fractions of a year.

After simplifying the integrals, DB_A_ becomes Eq. (3):

$${\text{DB}}_{\text{A}}=\frac{5}{6}t_{\text{O}}\left(1-q\left(t_{\text{O}}\right)\right)+\frac{1}{2}\left(t_{\text{R}}\right)\left[1-q\left(t_{\text{O}}\right)\right]+\left(t_{\text{A}}-t_{\text{R}}-t_{\text{O}}\right)\left[1-q\left(t_{\text{O}}+t_{\text{R}}\right)\right]$$ (3)

From this equation, we derived factors of 0.438 for a hospital case and 0.368 for an ambulatory case for calculating the DB over the entire acute phase as a function based on the lowest QoL and duration of the illness as a fraction of a year (see Supplemental Appendix Table 2 for detailed calculations). Equation (4a) gives the resulting disability estimates for hospitalized (hosp) cases and Eq. (4b) for ambulatory (ambu) cases.

$${\text{DB}}_{\text{A},\text{hosp}}=0.438\left(t_{\text{O}}+t_{\text{R}}\right)\left(1-q\left(t_{\text{0}}\right)\right)$$ (4a)

and

$${\text{DB}}_{\text{A},\text{ambu}}=0.368\left(t_{\text{O}}+t_{\text{R}}\right)\left(1-q\left(t_{0}\right)\right)$$ (4b)

### Disability-adjusted life years during the persistent symptoms phase.

**Figure 3.**
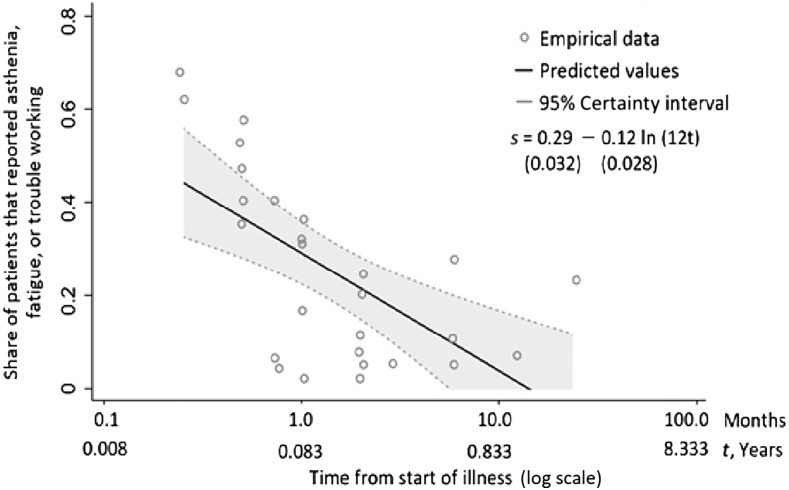
Share of patients experiencing persistent symptoms of dengue (adapted from Tiga et al.^8^). The share of patients experiencing persistent dengue is denoted by *s*, while *t* denotes the time from start of dengue illness measured in year. The numbers in parentheses below the equation are standard errors of the coefficients.

Having treated days 1 through 20 as the acute phase, we modeled the DALY burden due to persistent symptoms as the period beginning after the acute phase. A fraction of cases then experience persistent symptoms such as asthenia and fatigue. Examining the relationship between the share of patients with persistent symptoms and the time since the beginning of symptoms, Tiga et al.^8^ derived Eq. (5):

s(t)=0.29−0.12×ln(12t), (5)

where *s*(*t*) is the share of patients with persistent symptoms and *t* is the time from the first onset of illness (measured in years), and ln denotes the natural logarithm. According to Eq. (5), the share of patients with persistent symptoms at the end of the acute phase (20 days or 0.055 year) is 0.33. This share decreases over time after dengue onset, as shown in Figure 3. We use M to denote a patient’s maximal duration of the persistent symptoms phase. Based on Eq. (5), our best estimate of M is 0.93 year, when the share of patients with persistent symptoms reaches zero.

As the available literature does not distinguish variations in QoL within the persistent symptoms phase, we assumed that the level of disability or loss of QoL for subjects experiencing persistent symptoms was a constant over the persistent symptoms phase, denoted by dis_P_ Eq. (6) provides the basis for assessing the level of disability induced by persistent symptoms (DB_P_) from time A (0.055 years) days through M.

$${\text{DB}}_{\text{P}}=\int_{t_{\text{A}}}^{t_{\text{M}}}\left[s\left(t\right)\times {\text{dis}}_{\text{P}}\right]\text{dt}$$ (6)

After completing the integration, Eq. (6) became Eq. (7):

$${\text{DB}}_{\text{P}}=\left[\left(t_{\text{M}}-t_{\text{A}}\right)\left(0.29+0.12\right)-0.12\left(t_{\text{M}}\;\ln\left(12t_{\text{M}}\right)-t_{\text{E}}\;\ln\left(12t_{\text{E}}\right)\right)\right]\times {\text{dis}}_{\text{P}}$$ (7)

Salomon et al.^10^ report the most relevant estimate of the level of disability for post-acute effects (fatigue, emotional liability, and insomnia after an infectious disease episode). They estimated disability to be 0.219 (95% certainty interval [CI]: 0.148–0.308). We applied these values, as dis_p_, and estimated the impact of dengue on DALYs from persistent symptoms. Using Eq. (7), we estimated that the average DALYs caused by persistent symptoms over 11.2 months (*t*_M_) and applied this to all identified articles.

### Data analysis.

For each identified article with known information on QoL, on the worst day of a dengue illness episode, we calculated the area of a rectangle with height equal to the gap between the QoL at onset and that on the worst day, and width as the duration of the acute episode (as a fraction of a year). We then applied this area by the factors derived previously of 0.438 for a hospital case and 0.368 for an ambulatory case, to calculate the area above the QoL curve.

Some articles provided information for both hospital and ambulatory cases, some had only for hospital cases, while others had only for ambulatory cases. Given the limited number of articles, we examined the relationship of DALYs between hospital and ambulatory cases from articles that provided information on both settings, and then imputed the DALYs for the missing setting in the articles that did not report both hospitalized and ambulatory cases. For the acute phase, we calculated the mean, minimum, maximum, and standard deviation of DALYs from the selected literature review articles or abstracts, and generated the point estimate and range of DALYs. We combined the ambulatory and hospitalized settings by weighting using the 18% of dengue episodes hospitalized globally.^11^

We combined the acute and persistent symptoms phases to generate the overall DALYs from a dengue episode by setting, with medians, means and 95% CIs.

### Sensitivity analysis.

Our sensitivity analysis addressed four sources of the uncertainty. The first source related to the DALY burden estimated from the studies identified in the literature review for the acute phase. We assumed this burden followed a triangular distribution with the mean, minimum, and maximum estimated from the articles. The second source of uncertainty was the disability weight for persistent symptoms. Because disability weights need to be between zero and one, we assumed that they followed a beta distribution, the same distribution used for other chronic conditions in the latest guideline on cost-effectiveness analysis.^12^ We used the lower and upper bound of the 95% CI for the disability weight from Salomon et al.^10^ to estimate the lower and upper bound of DALYs due to persistent symptoms using Eq. (7). The third source of uncertainty related to the share of patients experiencing persistent symptoms at various follow-up times. The fourth source concerned the maximum duration of persistent symptoms represented by Eq. (5) from Tiga et al.^8^ We addressed the third and fourth uncertainty sources by obtaining the mean and variance-covariance matrix of the two coefficients (constant and slope terms) in Eq. (5) from the original data in Tiga et al.^8^ We randomly drew 1,000 sets of values from the joint distributions of the triangular distribution, the beta distribution, and the joint distribution of coefficients. For each draw in this simulation, we estimated the patient’s maximal duration of persistent symptoms (*t*_M_) and their probability of experiencing persistent symptoms from each time from *t*_A_ to *t*_M_. We then estimated the overall 95% CIs from the simulation results.

## RESULTS

**Figure 4.**
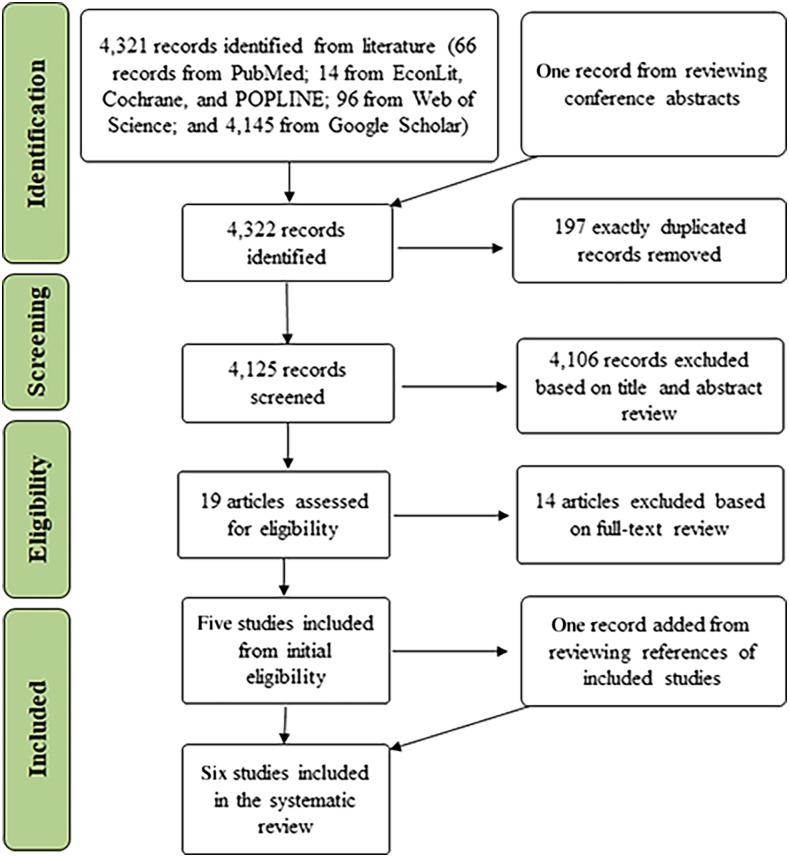
Preferred Reporting Items for Systematic Reviews and Meta-Analyses flow diagram for selection of empirical studies. This systematic review of original empirical studies (applicable only for the acute phase) initially identified 4,322 citations and finally included six studies. This figure appears in color at www.ajtmh.org.

Figure 4 presents the preferred reporting items for systematic reviews and meta-analyses flow diagram.^13^ It shows the numbers of studies identified and retained at subsequent stages of locating articles for review in this study. The initial search found 4,322 publications or abstracts. After removing duplicate articles and screening abstracts for relevance, we identified 19 potentially relevant publications for accessing the full text for review. Reviewing the text of these entries and the references of relevant studies, we retained the six studies that met our inclusion and exclusion criteria.^9,14–18^ The major reasons for exclusion during the full text review were: 1) studies did not measure QoL or disability (seven studies); 2) they were not empirical studies with primary data collection (four studies); 3) there was no full-text article available (one study); and 4) study results were duplicated (two studies).

Table 1 shows key characteristics of the six included studies. Their populations were from Brazil, Malaysia, Panama, Puerto Rico (one each), and Vietnam (two studies). All studies were conducted after 2000, with sample sizes of patients ranging from 64 to 372. Among the six studies, three had both hospitalized and ambulatory dengue cases, one had hospitalized dengue cases only, and two had ambulatory dengue cases only. A visual analogue scale, a widely used tool for the QoL assessment, was applied to the two studies in Vietnam and the studies in Malaysia and Panama. As all six studies achieved at least intermediate categories in the acceptability criteria, we considered all acceptable and included all of them in the subsequent analysis. We observed that all six studies reported only cases treated in the formal health sector.

**Table 1:** Characteristics of the six included studies

First author, last name (publication year)	Data years	Population and country	Sample size	Setting(s)	Cases included: inpatient or outpatient	Instrument to measure QoL or disability
Martelli (2011)	2005	Children and adults, Brazil	372	Hospitals with both inpatient and ambulatory settings	Both	World Health Organization QoL instrument
Lum (2008)	2004–2005	Children and adults, Malaysia	207	A medical center	Both	Visual analog scale
Armien (2008)	2005	Children and adults, Panama	162	Ambulatory public health facilities	Outpatient only	EuroQoL 100-point thermometer visual scale
Shepard (2017)	2010	Children and adults, Puerto Rico	101	Laboratory confirmed cases, hospitals and outpatient clinics	Both	EuroQol-5 dimension questionnaire
Nguyen (2013)	2010	Adults only, Vietnam	64	Hospital outpatient department	Outpatient only	Questionnaire and a visual analog scale
Whitehorn (2016)	2013–2015	Adults only, Vietnam	300	Two hospitals	Inpatient only	Visual analog scale

QoL = quality of life.

All six studies considered only the acute phase of the disease and only nonfatal cases. The average durations of illness were 13.1 and 12.3 days for ambulatory and hospitalized cases, respectively. Disability levels of the worst day were 0.62 and 0.71, and DALYs for the entire acute phase (first 20 days) were estimated as 0.0116 and 0.0159, for ambulatory and hospitalized cases, respectively (Table 2). Applying Eqs. (4a) and (4b), the hospitalized cases had slightly greater DALYs than ambulatory cases.

**Table 2:** DALY estimates from area above the quality-of-life curves shown in Figure 2 (acute phase only)

	Ambulatory cases			Hospitalized cases		
First author (last name)	Average duration of illness	Disability worst of day	DALYs	Average duration of illness	Disability of worst day	DALYs
Individual studies						
Martelli	10.9	0.80	0.0161	11.2	0.90	0.0216
Lum	9.5	0.56	0.0113	12.0	0.62	0.0148
Armien	20.0	0.67	0.0135	NA	NA	0.0181
Shepard	11.5	0.85	0.0164	14.2	0.84	0.0202
Nguyen	9.5	0.24	0.0047	NA	NA	0.0088
Whitehorn	NA	NA	0.0076	9.0	0.49	0.0118
Summary measures						
Mean	13.1	0.62	0.0116	12.3	0.71	0.0159
Standard error	2.0	0.11	0.0021	1.1	0.10	0.0022
Minimum	9.5	0.24	0.0047	9.0	0.49	0.0088
Maximum	20.0	0.85	0.0164	14.2	0.90	0.0216

DALYs = disability-adjusted life years; NA = not available. Average duration of illness is measured in days; the average DALY burden per episode was imputed based on the correlation between DALY burden for ambulatory and that for hospitalized cases.

Using Eq. (5) and the sensitivity analysis, the median (best estimate) of the share of patients experiencing symptoms at the end of 20 days is 0.338, duration of persistent symptoms was 0.087 years (1.04 months), the DALYs lost due to persistent symptoms was 0.019 DALYs, and the combined acute and persistent DALYs per episode are 0.031 for an ambulatory episode, 0.035 for a hospitalized episode, and 0.032 overall. The DALYs for the hospitalized episode were 14% higher than those for the ambulatory episode. Table 3 provides the overall distribution of the parameters. The distribution of the duration and associated burden is highly skewed.

**Table 3:** Results of sensitivity analyses (median, mean, and range)

Indicator	Median (best estimate)	Mean	95% Certainty interval
Share (*s*) of patients experiencing persistent symptoms after *t*_E_ (20 days)	0.3383	0.3379	0.2684–0.4084
DALYs from persistent symptoms	0.0192	0.0481	0.0080–0.0825
Overall duration of persistent symptoms	0.0865	0.2240	0.0401–0.3590
DALYs per episode (acute phase only)*			
Ambulatory episode	0.0107	0.0108	0.0057–0.0151
Hospitalized episode	0.0152	0.0153	0.0100–0.0201
Overall	0.0115	0.0116	0.0058–0.0185
Overall DALYs per episode†			
Ambulatory episode	0.0307	0.0588	0.0170–0.0917
Hospitalized episode	0.0351	0.0632	0.0241–0.0960
Overall	0.0315	0.0596	0.0178–0.0925

DALYs = disability-adjusted life years.

* Summing onset and recovery periods.

† Summing acute (onset and recovery periods) and persistent symptoms phases. All estimates are for nonfatal cases only.

**Figure 5.**
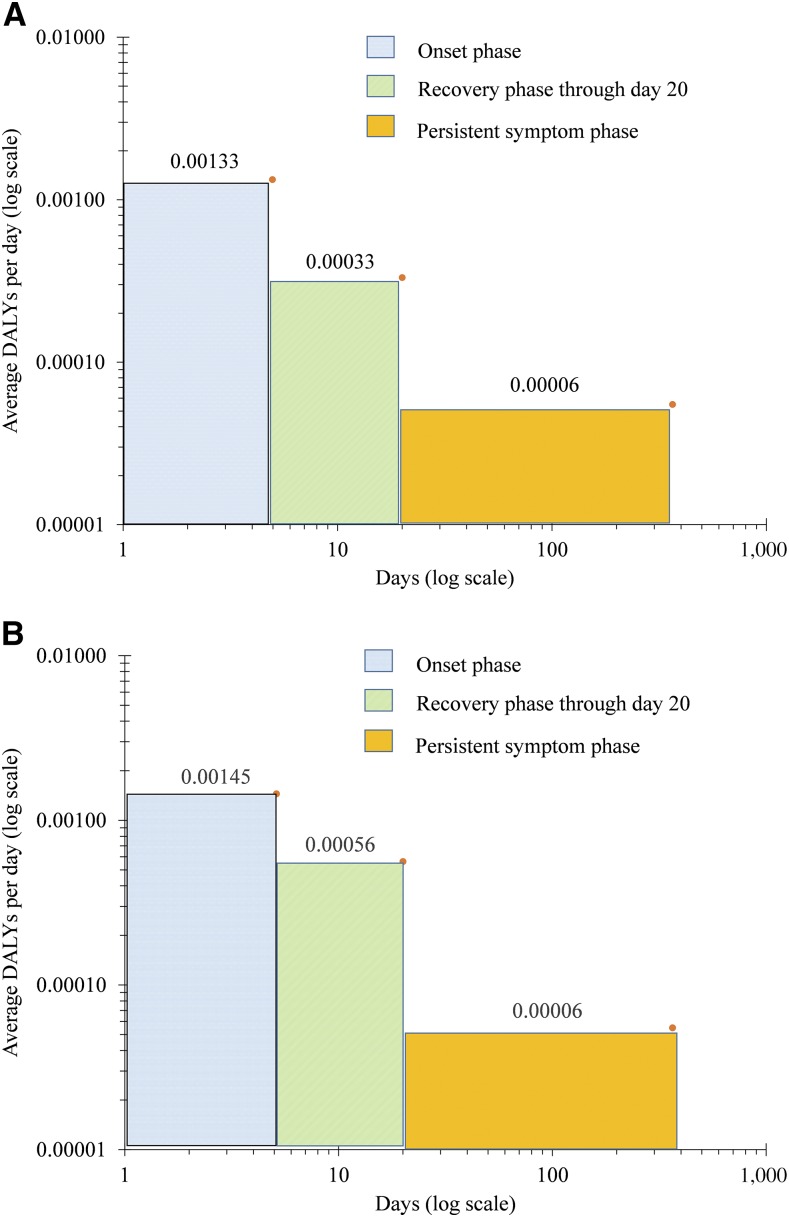
(**A**) Disability-adjusted life years (DALYs) per day of a nonfatal dengue episode by period for ambulatory cases. (**B**) Disability-adjusted life years per day of a nonfatal dengue episode by period for hospitalized cases. This figure appears in color at www.ajtmh.org.

Figure 5A for ambulatory cases and Figure 5B for hospitalized cases provide the DALYs (relative disability weights) per day for the three periods of a dengue episode. As expected, the onset period had the highest average DALY weight per day, and the average DALY weights declined as time passed. The contribution of each period to the total DALYs for an episode is height times width on linear (not log) scales. In terms of the contribution to the total DALYs per episode, the onset, recovery, and persistent symptoms period contributed 21.4%, 16.2%, and 62.4% in ambulatory cases, and 21.2%, 23.9%, and 54.9% in hospitalized cases, respectively.

## DISCUSSION

To our knowledge, this is the first systematic empirical analysis of DB per dengue episode. These estimates based on a systematic literature review generate several noteworthy findings. First, they show that the empirical information on the impact of an acute dengue episode on DALYs was limited. After carefully screening and reviewing articles, we found only six that included empirical data on DALYs and QALYs per dengue episode. Most economic evaluations of dengue interventions used DALYs or QALYs from secondary data (available literature). Second, the DALY weight for an acute hospitalized dengue episode on its worst day briefly places dengue among the most severe 2% of the 183 systematically assessed disease states.^10^ However, Figure 2 shows that this extreme severity lasts only a few days. Third, the DALYs for hospitalized episodes (0.035) were only slightly (14%) higher than for ambulatory episodes (0.031). Fourth, the DALYs for the acute phase varied substantially among the individual studies, from 0.0047 to 0.0163 for ambulatory cases and 0.0118 to 0.0195 for hospitalized cases (without considering imputed values). Fifth, because of the longer duration of the persistent symptoms phase, DALYs due to persistent symptoms are much higher than those due to acute symptoms.

Because of the fear that a dengue case could become life threatening, even some nonsevere dengue cases may be hospitalized as a precaution to ensure that definitive medical care, including precise fluid monitoring, is immediately available if needed. This behavior may explain why the DALY burden of a hospitalized episode was only 14% higher than that of an ambulatory episode.

For both ambulatory and hospitalized episodes, the majority of DALY burden was due to the persistent symptoms phase. Because of the uncertainty around the persistent symptoms phase, however, the 95% CI was wide with the upper bound being 5.1 times the lower bound. We thus consider the median values more stable and representative.

This review has synthesized studies examining QoL loss only for dengue patients. In reality, the illness also affects QoL for the patient’s entire household. An eight-country study by Suaya^19^ quantified this impact as the “days affected” by dengue within the household. Economically, the impact of dengue on households is also substantial. Indirect costs shouldered by households represent a significant share of global dengue economic costs.^11^ The fear that a dengue episode could become life threatening likely contributes to household burden.

Our best estimate of DALYs per case (0.032) is more than three times that of the 2013 GBD’s approximation of the DB of a dengue episode (0.0097). On an aggregate basis, the GBD study estimated a total of 1.14 million DALYs were due to dengue in 2013, of which half resulted from premature mortality and half due to morbidity.^2^ Assuming that DALYs due to premature death remain the same and hospitalized and ambulatory dengue cases account for 18% and 82%, respectively,^11^ using our estimates of DALYs per episode would result in a total of 2.4 million DALYs due to dengue, which is 2.1 times the 2013 GBD estimate.^2^ Similarly, because of the potential underestimation of DALYs per dengue case, the cost-effectiveness of interventions to address dengue illness might also be underestimated. A strength of our analysis is that all estimates for the acute portion were specific for dengue, whereas GBD used a generic value for acute infectious diseases. Global Burden of Diseases’ undervaluation of dengue burden could affect the global resource allocation to combat this condition.

The GBD study that reported 89.9% of years of life lost because of disability from dengue were from chronic fatigue.^2^ Although not quite as high, our study also found that the DB from persistent symptoms was substantial, accounting for 62.4%, 54.9%, and 61.0% of total DALYs for ambulatory, hospitalized, and overall cases, respectively. Thus, the many studies that consider only the acute portion of a dengue episode may substantially underestimate the overall burden.

Several limitations of this study must be acknowledged. First, only six studies met our inclusion criteria for this review and QoL varied substantially among countries. Thus, our estimated means and ranges of DALYs per episode may change if more data become available. Second, we used Lum’s study^9^ to derive the factors to calculate DALYs for the acute phase based on the QoL on the worst day and duration of each period. As we found no other report of QoL by day, we could not validate the generalizability of Lum’s study. Third, we used the regression model to impute DALYs for articles that did not include data for either hospital or ambulatory cases. Given only four studies with data from both settings, additional data could change the imputations. Fourth, we considered the ambulatory cases in these studies as representative of all ambulatory dengue cases. Fifth, none of the studies examined ambulatory cases outside the formal medical system, such as those self-treated or seen by a traditional healer. Consistent with previous estimates of their indirect costs,^11^ we assumed that the DALY burden of these cases was comparable with that of ambulatory cases treated in the formal health system. Because of these limitations, our estimated DALY burden of a nonfatal dengue episode might be might be too high. Sixth, although persistent dengue constituted most of the DALY burden, there is substantial uncertainty around that phase’s prevalence and duration. Despite these limitations, this study summarized current knowledge of DALYs from dengue, provided the point estimate and the uncertainty around the impact of a dengue episode on DALYs, an indicator used widely in disease burden studies and cost-effectiveness analysis. In addition, this study also highlighted the need for more primary studies concerning dengue episodes on quality of care and disability to provide more accurate estimates for both disease burden of dengue and cost-effectiveness analysis of interventions addressing dengue. Studies with follow-up of a year or more would be particularly valuable, as they would inform the rates of persistent dengue and might identify actions to mitigate or prevent it.

Refining the estimate of the impact of dengue episodes on DALYs would help in prioritizing interventions to address dengue illnesses and would inform prioritization and cost-effectiveness studies. Disability-adjusted life year or QALY weights are vital for conducting cost-effectiveness analyses of dengue interventions to address dengue burden. In addition, these new estimates would also help to ensure DALYs per dengue episode are estimated consistently. Consistency is an important goal of disability measures, allowing for objective comparison of dengue against other health problems.

## Supplementary Material

Supplemental appendix
