# Supplementary material for: Impact of a Nonfatal Dengue Episode on Disability-Adjusted Life Years: A Systematic Analysis
Source: Am J Trop Med Hyg. 2018 Oct 1;99(6):1458–65. doi: 10.4269/ajtmh.18-0309 (PMC6283510; doi:10.4269/ajtmh.18-0309)
Supplement: Supplementary file 1 [file tpmd180309.SD1.pdf]

Supplemental Appendix

*Supplemental Appendix Table A1. Acceptability assessment of identified articles*

| Categories | Choices |
|---|---|
| Representative-ness | 1. Sample universe clearly represents a group of dengue patients defined by severity or treatment setting (100%)<br>2. Intermediate or not clear (50%)<br>3. Sample cannot be related to any specific severity or treatment setting (0%) |
| Sampling | 1. Random sampling (multi-stage random sampling) (100%)<br>2. Cluster random sampling (67%)<br>3. Unclear (33%)<br>4. Convenience sampling (0%) |
| Sample size | 1. Adequate (relates to the precision for estimating average disability in the reference population) (100%)<br>2. Uncertain (50%)<br>3. Not acceptable (0%) |
| Sample losses from initial sampling | 1. 0-4% of sample (100%)<br>2. 5-9% of sample (67%)<br>3. 10-19% of sample (33%)<br>4. 20% or more of the sample (0%) |
| Measurement of DALYs or QALYs | 1. Measured with standardized instrument (100%)<br>2. Measured with partially standardized instrument (67%)<br>3. Measured with self-developed instrument (33%)<br>4. Measured with no instrument (e.g. expert opinion) (0%) |
| Appropriateness of statistical analysis | 1. Appropriate (100%)<br>2. Uncertain (50%)<br>3. Not appropriate (0%) |

Notes: DALYs denotes disability-adjusted life years; QALYs denotes quality-adjusted life years

*Supplemental Appendix Table A2. Derivation of factors to estimate DALYs for the acute phase using Lum et al.*[9]

| Components* | Hosptalized | | | | Ambulatory | |
|---|---|---|---|---|---|---|
| | Adults | | Children | | | |
| | With Leakage | No leakage | With Leakage | No leakage | Adults | Children |
| Lowest quality of life, as decimal, (a) | 0.37 | 0.36 | 0.40 | 0.38 | 0.44 | 0.44 |
| Highest disability, as decimal, (b) = 1 - (a) | 0.63 | 0.64 | 0.60 | 0.62 | 0.56 | 0.56 |
| Lowest day, (c) | 5 | 5 | 5 | 3 | 5 | 5 |
| Duration of days (reported), (d) | 13.6 | 12.6 | 12.8 | 10.3 | 10.2 | 8.8 |
| Recovery (days), (e ) = (d) - (c ) | 8.6 | 7.6 | 7.8 | 7.3 | 5.2 | 3.8 |
| Onset product, (f) = (b) x (c) | 3.15 | 3.2 | 3 | 1.86 | 2.8 | 2.8 |
| Onset product/365, (g) = (f) / 365 | 0.00863 | 0.00877 | 0.00822 | 0.00510 | 0.00767 | 0.00767 |
| (5/6) Onset product, (h) = 5/6 x (g) | 0.00719 | 0.00731 | 0.00685 | 0.00425 | 0.00639 | 0.00639 |
| Recovery product, (i) = (b) x (c) | 5.418 | 4.864 | 4.68 | 4.526 | 2.912 | 2.128 |
| Recovery product/365 (j) = (i) / 365 | 0.01484 | 0.01333 | 0.01282 | 0.01240 | 0.00798 | 0.00583 |
| (1/2) Recovery product, (k) = 1/2 x (j) | 0.00742 | 0.00666 | 0.00641 | 0.00620 | 0.00399 | 0.00292 |
| Remaining days (20-duration), (l) = 20 - (d) | 6.4 | 7.4 | 7.2 | 9.7 | 9.8 | 11.2 |
| Disability weight during remaining days, (m) | 0.06 | 0.08 | 0.05 | 0.00 | 0.05 | 0.05 |
| Remaining product, (n) = (l) x (m) | 0.384 | 0.592 | 0.360 | 0.000 | 0.490 | 0.560 |
| Remaining product/365, (o) = (n) / 365 | 0.00105 | 0.00162 | 0.00099 | 0.00000 | 0.00134 | 0.00153 |
| Onset+recovery+remaining, (p) = (h) + (k) + (o) | 0.01567 | 0.01559 | 0.01425 | 0.01045 | 0.01172 | 0.01084 |
| Number of observations, (q) | 49 | 26 | 44 | 3 | 75 | 8 |
| Observation days, (r) | 20 | 20 | 20 | 20 | 20 | 20 |
| Area of rectangle, (s) = (b) x 20 / 365 | 0.0345 | 0.0351 | 0.0329 | 0.0340 | 0.0307 | 0.0307 |
| Factor (area above curve/rectangle), (t) = (p) / (s) | 0.4538 | 0.4446 | 0.4333 | 0.3075 | 0.3821 | 0.3533 |
| Overall burden weighted by leakage, (u) | 0.01564 | | 0.01400 | | 0.01172 | 0.01084 |
| Factor weighted by patients with leakage, (v) | 0.4506 | | 0.4253 | | 0.3821 | 0.3533 |
| Average burden of adults and children, (w) | 0.01482 | | | | 0.01128 | |
| Average factor of adults and children, (x) | 0.4380 | | | | 0.3677 | |

*In rows (u) and (v), the burden and factors are weighted averages of entries in rows (p) and (t), respectively. The weights were derived from (q), the number of observations of dengue hospitalized cases with leakage and without leakage. For adults, weights of 49/75 and 26/75 were assigned to hospitalized dengue cases with and without leakage, respectively. For children, weights of 44/47 and 3/47 were assigned to hospitalized dengue cases with and without leakage, respectively. In rows (w) and (x), the burden and factors are the simple averages of values in rows (u) and (v), respectively, for adults and children.
